# Differences in primary molecular subtype distribution contribute to racial and ethnic disparities in adult-type diffuse gliomas

**DOI:** 10.1093/neuonc/noag157

**Published:** 2026-07-14

**Authors:** David Gomez, Ishan Shah, David J Cote, Mackenzie Price, Julia R Benedetti, John Pham, Carol Kruchko, Robert G Briggs, Gabriel Zada, Quinn T Ostrom

**Affiliations:** Department of Neurosurgery, Keck School of Medicine, University of Southern California, Los Angeles, California, USA; Department of Neurosurgery, Keck School of Medicine, University of Southern California, Los Angeles, California, USA; Department of Neurosurgery, Keck School of Medicine, University of Southern California, Los Angeles, California, USA; Central Brain Tumor Registry of the United States, Hinsdale, Illinois, USA; Central Brain Tumor Registry of the United States, Hinsdale, Illinois, USA; Department of Neurosurgery, Duke University School of Medicine, Durham, North Carolina, USA; Department of Neurosurgery, Warren Alpert School of Medicine, Brown University, Providence, Rhode Island, USA; Central Brain Tumor Registry of the United States, Hinsdale, Illinois, USA; Department of Neurosurgery, Keck School of Medicine, University of Southern California, Los Angeles, California, USA; Department of Neurosurgery, Keck School of Medicine, University of Southern California, Los Angeles, California, USA; Central Brain Tumor Registry of the United States, Hinsdale, Illinois, USA; Department of Neurosurgery, Duke University School of Medicine, Durham, North Carolina, USA; The Preston Robert Tisch BT Center, Duke University School of Medicine, Durham, North Carolina, USA; Duke Cancer Institute, Duke University Medical Center, Durham, North Carolina, USA (Q.T.O.)

**Keywords:** biomarkers, central brain tumor registry, disparity, glioma

## Abstract

**Background:**

Molecular profiling has become an integral part of glioma classification. The extent to which the incidence of molecularly defined adult-type gliomas varies by demographics is unknown. We describe national-level incidence and overall survival patterns of selected glioma subtypes by race/ethnicity.

**Methods:**

We generated standardized average annual age-adjusted incidence rates of molecularly defined glioma subtypes by race/ethnicity from the Central Brain Tumor Registry of the United States (CBTRUS) using newly diagnosed cases from January 1, 2018, to December 31, 2022. Survival data from the National Cancer Database (NCDB) were used from newly diagnosed cases from January 1, 2018, to December 31, 2021 (with follow-up through December 31, 2022) to evaluate 4-year overall survival, median survival, and multivariable Cox proportional hazards ratios.

**Results:**

CBTRUS identified 68 172 glioma cases; IDH-wild-type glioblastoma was most common (*n* = 51 548). Non-Hispanic White individuals had a significantly higher incidence of all gliomas compared to other groups (*P*  > .001). Non-Hispanic Black individuals had the lowest incidence for IDH-mutant astrocytoma and oligodendroglioma, and shared lowest incidence of IDH-mutant glioblastoma and IDH-wild-type astrocytoma with non-Hispanic other individuals, who had the lowest incidence of IDH-wild-type glioblastoma (*P* < .001). The odds of having an IDH-mutant (versus IDH-wildtype) astrocytoma or glioblastoma were significantly lower for males, those of older age, and non-Hispanic Black individuals (*P* < .001). Non-Hispanic other individuals also had increased adjusted overall survival for most glioma subtypes compared to other racial/ethnic groups (*P* < .001). Four-year overall survival was lowest in all racial/ethnic groups for IDH-wild-type glioblastoma (*P* < .001).

**Conclusions:**

Our findings reveal significant disparities in incidence and survival by race/ethnicity with notable variations present based on glioma biomarkers.

Key Points

- In this epidemiological study from the Central Brain Tumor Registry of the United States, we find significant disparities by race/ethnicity in the incidence and survival of cases diagnosed with a glioma stratified by clinically relevant biomolecular markers.
- In particular, we find increased incidence and worse survival for non-Hispanic White cases across different glioma subtypes.

Importance of the Study

This study underscores the complexity and value of molecular glioma subtyping for improved risk assessment in diverse patient populations, revealing significant disparities in incidence and survival by race/ethnicity with notable variations present based on glioma biomarkers.

Gliomas account for 22.2% of all primary brain and central nervous system tumors in the United States; glioblastoma, the most common glioma subtype, represents 52.2% of all malignant brain tumors.^1-3^ Historically, classification relied on cellular differentiation; however, advancements in molecular genetics have since revolutionized the understanding, classification, and management of these tumors. Landmark clinical trials have shown the impact of key molecular alterations such as isocitrate dehydrogenase 1/2 (*IDH1/2*) mutations, O6-methylguanine-DNA methyltransferase (*MGMT*) promoter methylation, and co-deletion of the p arm of chromosome 1 and the q arm of chromosome 19 (1p/19q co-deletion), which correlate with enhanced sensitivity to alkylating chemotherapy and radiation, leading to more favorable survival.^4-7^ Consequently, the 2021 World Health Organization (WHO) classification now characterizes gliomas by molecular features, with IDH-wild-type status, for instance, defining glioblastoma.^8^ Accordingly, routine testing and management are now driven by a glioma’s unique genetic profile.^9-11^ Despite these advancements, glioblastoma remains one of the most fatal cancers with a recent estimation of median survival at 12.4 months.^12^

Decades of population-level research demonstrate persistent disparities in glioma incidence and outcomes by race and ethnicity. Non-Hispanic White individuals, for example, have exhibited both higher incidence and lower survival across most glioma subtypes, even when adjusting for socioeconomic status (SES) and variations in treatment.^13-17^ One proposed theory posits underlying variance in prevalence of specific molecular subtypes between racial/ethnic groups, but population-level evidence incorporating such molecular profiling has remained limited. More recent work reveals additional molecular distinctions by race and ethnicity, including an observed enrichment of IDH-wild-type glioblastoma in non-Hispanic White individuals and a higher prevalence of IDH mutations in glioblastomas from Hispanic cases of Mexican ancestry compared to US Hispanic cases.^18^^,^^19^ Starting in 2018, US cancer registries began collecting and reporting molecular data for selected brain tumor subtypes, aggregating this information using a combination of International Classification of Diseases for Oncology, 3rd edition (ICD-O-3) codes and the “Brain Molecular Markers” (BMM) site-specific data item. Initial reports from the first diagnosis year confirm overall lower survival with IDH-wild-type versus IDH-mutant gliomas.^1^^,^^3^^,^^20^ However, detailed investigation into these differences by race and ethnicity using these population-level molecular data has been limited by its recency.

Now that multiple years of national molecular data are available, this study aimed to address this critical gap in understanding how race and ethnicity relate to glioma incidence and survival by leveraging newly available multi-year incidence data from the Central Brain Tumor Registry of the United States (CBTRUS) and survival data from the National Cancer Database (NCDB) for the most common molecularly defined adult-type diffuse glioma subtypes. This work aims to investigate how historical disparities persist or change when gliomas are defined by their molecular profiles, offering timely insights to better inform future research and ­clinical efforts in the current molecular era.

## Methods

### Ethical Approval and Data Sources

This study utilized anonymized population-based data and received exempt approval from the Duke University Institutional Review Board. For descriptive epidemiology and incidence analyses (diagnosis years 2018-2022), CBTRUS data were used. All incidence rates are presented per 100 000 population and age-adjusted to the 2000 US standard population. CBTRUS combines data from the Centers for Disease Control and Prevention’s (CDC) National Program of Cancer Registries (NPCR) and the National Cancer Institute’s (NCI) Surveillance, Epidemiology, and End Results (SEER) Program, representing the entire US population.^1^ Due to molecular data limitations within CBTRUS for survival outcomes, survival analyses were conducted using the NCDB for individuals diagnosed between January 1, 2018, and December 31, 2021, with follow-up through December 31, 2022. The NCDB includes data submitted from hospital tumor registries from over 1500 Commission on Cancer-accredited facilities and includes approximately 85% of new malignant brain tumor diagnoses in the United States and Puerto Rico.^21^

### Study Population

Molecular classification used the BMM site-specific data element (North American Association of Central Cancer Registries [NAACCR] Item #3816) and corresponding ICD-O-3 codes to identify IDH-mutation and 1p/19q co-deletion status. The *WHO Classification of Tumors of the Central Nervous System* included biomarkers in its 2016 revision, with additional changes made to the 2021 revision including the removal of IDH-wild-type astrocytoma and IDH-mutant glioblastoma subtypes. However, implementing the collection of these markers in cancer registration is multi-faceted and takes time. The 2021 WHO classification changes began reporting with cases diagnosed starting January 1, 2024, and will not be available for analysis by CBTRUS until 2027, as cancer registry public data release lags about 3 years behind. Additionally, these codes are still used by some pathologists and get coded in cancer registration.

The analysis included 5 molecularly defined subtypes: IDH-mutant astrocytoma (ICD-O-3: 9400/3, 9401/3, BMM: 01, 02), IDH-wild-type astrocytoma (ICD-O-3: 9400/3, 9401/3, BMM: 03, 04), IDH-mutant and 1p/19q co-deleted oligodendroglioma (ICD-O-3: 9450/3, 9451/3, BMM: 06, 07), IDH-wild-type glioblastoma (ICD-O-3: 9440/3, BMM: 05), and IDH-mutant glioblastoma (ICD-O-3: 9445/3). All individuals with a histopathologically confirmed adult-type diffuse glioma corresponding to a histology code for which molecular marker data were collected were included. All eligible individuals meeting these criteria within CBTRUS and NCDB databases for the specified time periods were included in the respective analyses.

Hispanic ethnicity was defined using the NAACCR Hispanic/Latino Identification Algorithm (v2.2.1), which classifies records as Hispanic or non-Hispanic based on registry fields (Spanish/Hispanic origin, birthplace, race, surnames). Race in US cancer registries is abstracted from patient clinical records or self-reported. Final group classifications included non-Hispanic White, non-Hispanic Black, Hispanic (all races), and non-Hispanic other (non-Hispanic American Indian/Alaska Native and non-Hispanic Asian or Pacific Islander) individuals. Overall, 1% of cases were missing race and/or ethnicity data and were excluded from incidence and survival analysis.

### Statistical Analysis

Urbanicity was defined by the county using 2013 Rural-Urban Continuum Codes (RUCCs) as Metropolitan (RUCCs = 1-3) or non-Metropolitan (RUCCs = 4-9). WHO grade at diagnosis was reassigned based on the 2025 CBTRUS glioma grade recode which used the 2021 WHO classification to define grade by glioma histology using the Collaborative Stage Site-Specific Factor 1 - WHO Grade Classification for cases diagnosed 2011-2017, and grade pathological or grade clinical for cases diagnosed after 2017 (see Price et al^1^ for additional information). Differences in proportions by BMM subtype were assessed using Pearson’s chi-squared tests and Cohen’s *w* effect size. Cohen’s *w* is a measure of the average difference divided by the standard deviation of the change. Conventional interpretation of Cohen’s *w* considers 0.00-0.10 to be negligible, 0.10-0.30 to be small, 0.30-0.50 to be medium, and 0.50 or more to indicate a large effect size. Differences in categorical age by BMM subtype were assessed using a 1-sided ANOVA test and partial eta-squared effect size. Multivariable logistic regression was used to estimate odds ratios (OR) and 95% CI of IDH mutation (reference = IDH-wild-type) among all glioblastomas and astrocytomas and differences in WHO grades 2 (reference) and 3 among IDH-mutant astrocytomas and oligodendrogliomas. All regression models were adjusted for sex, age at diagnosis, race and ethnicity group, and urbanicity. Average age-adjusted incidence rates (AAAIRs) and incidence rate ratios (IRRs) were generated using SEER*Stat (version 8.4.4).^22^ Univariable and multivariable Cox proportional hazards models were performed to estimate hazard ratios (HRs) with 95% CIs and *P*-values. Only those variables with a significant *P*-value <.05 were included in the multivariable Cox proportional hazards models. Individuals were right-censored at the time of their last follow-up, and the median overall follow-up was estimated using reverse Kaplan-Meier. Grade 1 and grade 4 oligodendroglioma, not otherwise specified (NOS) (ICD-O-3 histology code 9450/3), were excluded from all grade-stratified analyses. Four-year overall and median survival, along with adjusted survival curves, are presented per group stratified by race/ethnicity. Pairwise analyses of race and ethnicity groups were done on all multivariable (Tukey-adjusted) and hazards (Holm-adjusted) analyses. Statistical significance was set at *P* < .05. Analyses and figure generation were conducted using R (version 4.3.1).^23^

## Results

### Demographic and Clinical Characteristics

From 2018 to 2022, a total of 68 172 histopathologically confirmed, molecularly defined glioma cases with available race and Hispanic ethnicity data were reported in CBTRUS (Table 1 and Table S1). Overall, IDH-wild-type glioblastoma was the predominant diagnosis (76%), while IDH-mutant glioblastoma was the least common (2.1%). Non-Hispanic White individuals comprised most of the total cohort (80%) and were, on average, approximately 7-9 years older (Cohen’s *w* = 0.02, *P* < .001). Hispanic cases comprised 9.6% of the cohort, followed by non-Hispanic Black (6.2%) and non-Hispanic other cases (3.8%). There was a male predominance across all molecularly defined glioma diagnoses (Cohen’s *w* = 0.02, *P* < .001; Table S1) and when stratified by race and ethnicity (Cohen’s *w* = 0.02, *P* < .001).

**Table 1.** Descriptive table of histopathologically confirmed, molecularly defined glioma by race and hispanic ethnicity for diagnosis years 2018-2022

	Non-Hispanic White, *N* = 54 787^a^	Hispanic (all races), *N* = 6576^a^	Non-Hispanic Black, *N* = 4247^a^	Non-Hispanic Other, *N* = 2562^a^	Overall, *N* = 68 172^a^	Effect size	*P*-value
Molecularly defined glioma^b^						0.04	**<.001**
Astrocytoma, IDH-mutant (9400/3 and 9401/3)	4645 (8.5%)	766 (12%)	410 (9.7%)	274 (11%)	6095 (8.9%)		
Astrocytoma, IDH-wild-type (9400/3 and 9401/3)	3422 (6.2%)	414 (6.3%)	304 (7.2%)	181 (7.1%)	4321 (6.3%)		
Glioblastoma, IDH-wild-type (9440/3)	42 019 (77%)	4574 (70%)	3205 (75%)	1750 (68%)	51 548 (76%)		
Glioblastoma, IDH-mutant (9445/3)	1120 (2.0%)	169 (2.6%)	102 (2.4%)	69 (2.7%)	1460 (2.1%)		
Oligodendroglioma, IDH-mutant and 1 p/19q co-deleted (9450/3 and 9451/3)	3581 (6.5%)	653 (9.9%)	226 (5.3%)	288 (11%)	4748 (7.0%)		
Age at diagnosis^c^	64 (53, 72)	57 (44, 68)	59 (48, 68)	58 (44, 68)	63 (51, 71)	0.02	**<.001**
Sex						0.02	**<.001**
Female	22 882 (42%)	2850 (43%)	1902 (45%)	1123 (44%)	28 757 (42%)		
Male	31 905 (58%)	3726 (57%)	2345 (55%)	1439 (56%)	39 415 (58%)		
Radiation treatment^b^						0.03	**<.001**
No radiation/unknown	53 356 (97%)	6411 (97%)	4211 (99%)	2483 (97%)	66 461 (97%)		
Received any radiation	1431 (2.6%)	165 (2.5%)	36 (0.8%)	79 (3.1%)	1711 (2.5%)		
Age at diagnosis group^b^						0.11	<0.001
<50	11 061 (20%)	2201 (33%)	1166 (27%)	852 (33%)	15 280 (22%)		
50+	43 726 (80%)	4375 (67%)	3081 (73%)	1710 (67%)	52 892 (78%)		
Extent of surgical resection^b^						0.02	**<.001**
Gross total resection	22 826 (42%)	2653 (40%)	1728 (41%)	1026 (40%)	28 233 (41%)		
No surgery or biopsy only	16 036 (29%)	1769 (27%)	1267 (30%)	704 (27%)	19 776 (29%)		
Other or unknown surgery	389 (0.7%)	69 (1.0%)	—	<16 cases	500 (0.7%)		
Subtotal resection	15 536 (28%)	2085 (32%)	—	—	19 663 (29%)		
Urbanicity of county of residence^b^						0.12	**<.001**
Metropolitan	43 556 (83%)	5971 (95%)	3855 (92%)	2248 (93%)	55 630 (85%)		
Non-Metropolitan	8760 (17%)	328 (5.2%)	325 (7.8%)	178 (7.3%)	9591 (15%)		
Unknown	2471	277	67	136	2951		
WHO grade^b^ ^,^ ^d^						0.03	**<.001**
Unknown/other	767 (1.3%)	113 (1.7%)	81 (1.9%)	65 (2.5%)	1026 (1.5%)		
WHO Grade 2	6011 (11%)	1020 (16%)	495 (12%)	393 (15%)	7919 (12%)		
WHO Grade 3	4907 (9.0%)	708 (11%)	368 (8.7%)	290 (11%)	6273 (9.2%)		
WHO Grade 4	43 102 (79%)	4735 (72%)	3303 (78%)	1814 (71%)	52 954 (78%)		

Counts and rates are not presented when fewer than 16 cases were reported for the specific category. The suppressed cases are included in the counts and rates for totals. Bold denotes statistical significance.

Abbreviation: WHO, World Health Organization.

Data Source: Central Brain Tumor Registry of the United States Statistical Report: US Cancer Statistics—Centers for Disease Control and Prevention’s National Program of Cancer Registries and National Cancer Institute’s Surveillance, Epidemiology, and End Results Program, 2018-2022.

a *n* (%).

b Cohen’s *w* effect size and Pearson’s chi-squared test.

c Median (Q1, Q3), partial η² effect size and 1-sided ANOVA test.

d Grade was recoded using the CBTRUS glioma grade recode (see Price et al^1^ for additional information). https://doi.org/10.1093/neuonc/noaf194.

### Incidence of Molecularly Defined Gliomas by Race and Ethnicity

In all molecularly defined glioma subtypes, incidence was significantly higher for non-Hispanic White populations compared to other racial/ethnic groups (*P* < .001 for all; Figure 1; Table S2). Among minority populations, significant heterogeneity in glioma incidence was observed. For IDH-wild-type glioblastoma, Hispanic individuals (AAAIR: 1.83; 95% CI: 1.78-1.89) demonstrated higher incidence than both non-Hispanic Black (AAAIR: 1.342; 95% CI: 1.37-1.47) and non-Hispanic other individuals (AAAIR: 1.36; 95% CI: 1.29-1.42). Similarly, for IDH-mutant astrocytoma, incidence was higher in Hispanic individuals (AAAIR: 0.24; 95% CI: 0.22-0.25) compared to non-Hispanic Black individuals (AAAIR: 0.19; 95% CI: 0.17-0.21). For IDH-mutant and 1p/19q co-deleted oligodendrogliomas, non-Hispanic Black individuals (AAAIR: 0.11; 95% CI: 0.09-0.12) showed lower incidence than both non-Hispanic other (AAAIR: 0.22; 95% CI: 0.20-0.25) and Hispanic individuals (AAAIR: 0.21; 95% CI: 0.19-0.23). In contrast, we did not observe statistically significant differences in the incidence rates for IDH-wild-type astrocytomas and IDH-mutant glioblastomas among non-Hispanic Black, Hispanic, and non-Hispanic other cases.

**Figure 1.**
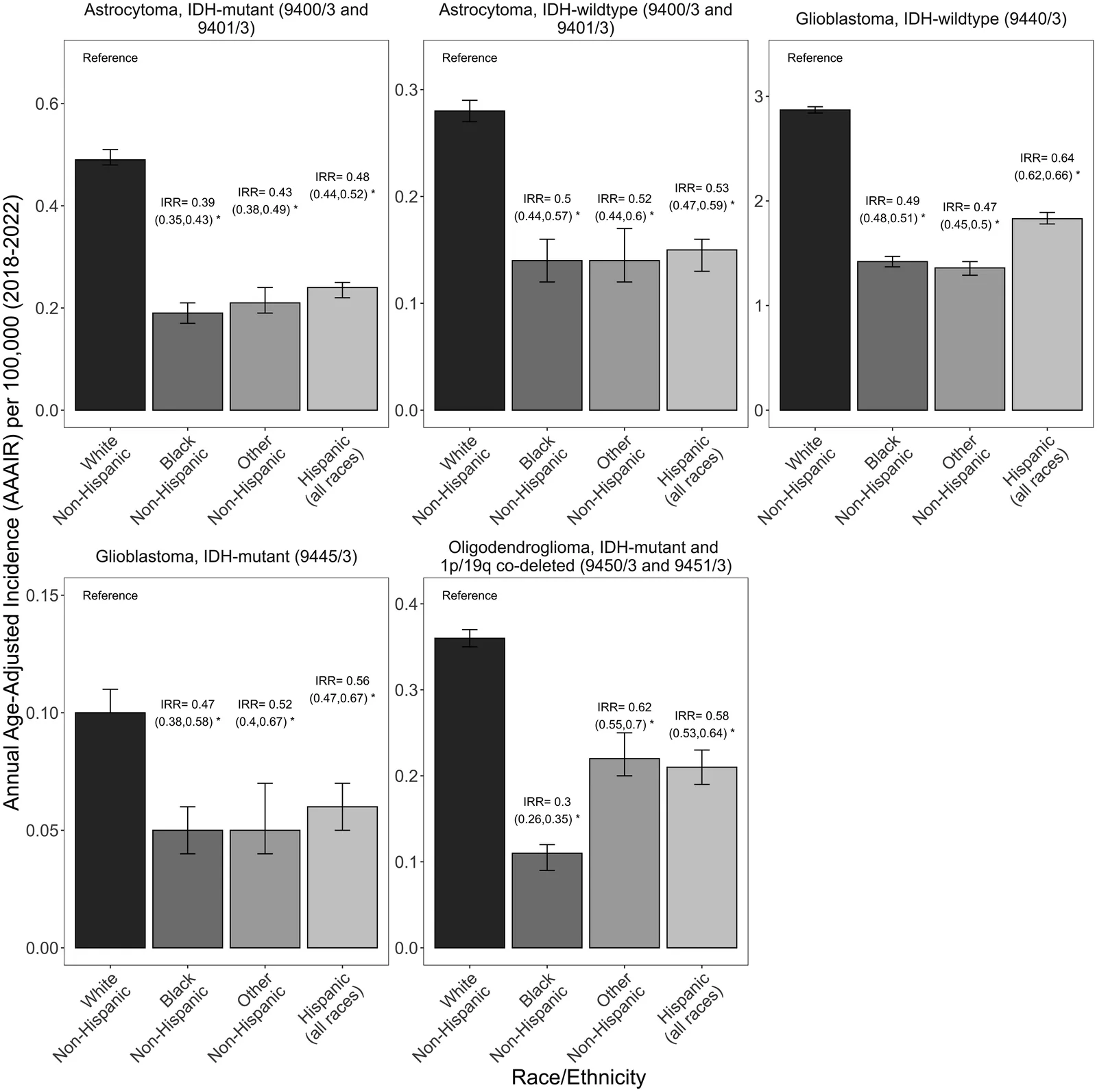
Average annual age-adjusted incidence rates^a^ and incidence rate ratios (IRR) with 95% confidence intervals of histopathologically confirmed, molecularly defined brain and other central nervous system glioma by race and ethnicity for diagnosis years 2018-2022. ^a^Rates are per 100 000 and are age-adjusted to the 2000 US standard population. Non-Hispanic White used for the denominator (reference) in IRR calculations. *Significant results with *P*-value < .05. Data Source: Central Brain Tumor Registry of the United States Statistical Report: US Cancer Statistics—CDC’s National Program of Cancer Registries and NCI’s Surveillance, Epidemiology, and End Results Program, 2018-2022.

### Associations With IDH-Mutation Status and WHO Grade

IDH-mutation varied by subtype and racial/ethnic group (Figure S1). Multivariable regression models were used to identify demographic factors associated with IDH-mutation status among astrocytomas and glioblastomas (Figure 2A). Non-Hispanic Black individuals had 23% significantly lower odds of having an IDH-mutant tumor compared to non-Hispanic White individuals (OR: 0.77; 95% CI: 0.69-0.86; *P* < .001). Male sex was also associated with significantly lower odds of an IDH-mutant tumor (OR: 0.90; 95% CI: 0.852-0.95; *P* < .001), as was increased age at diagnosis (OR: 0.92; 95% CI: 0.92-0.92, *P* < .001). There was insufficient statistical evidence to detect a difference in the odds of IDH mutation for Hispanic or non-Hispanic other cases or by urbanicity. In pairwise comparisons across all racial and ethnic groups, non-Hispanic Black individuals had significantly lower odds of having an IDH-mutant tumor than non-Hispanic White, Hispanic, and non-Hispanic other individuals, with no other pairwise differences reaching statistical significance (Table S3).

**Figure 2.**
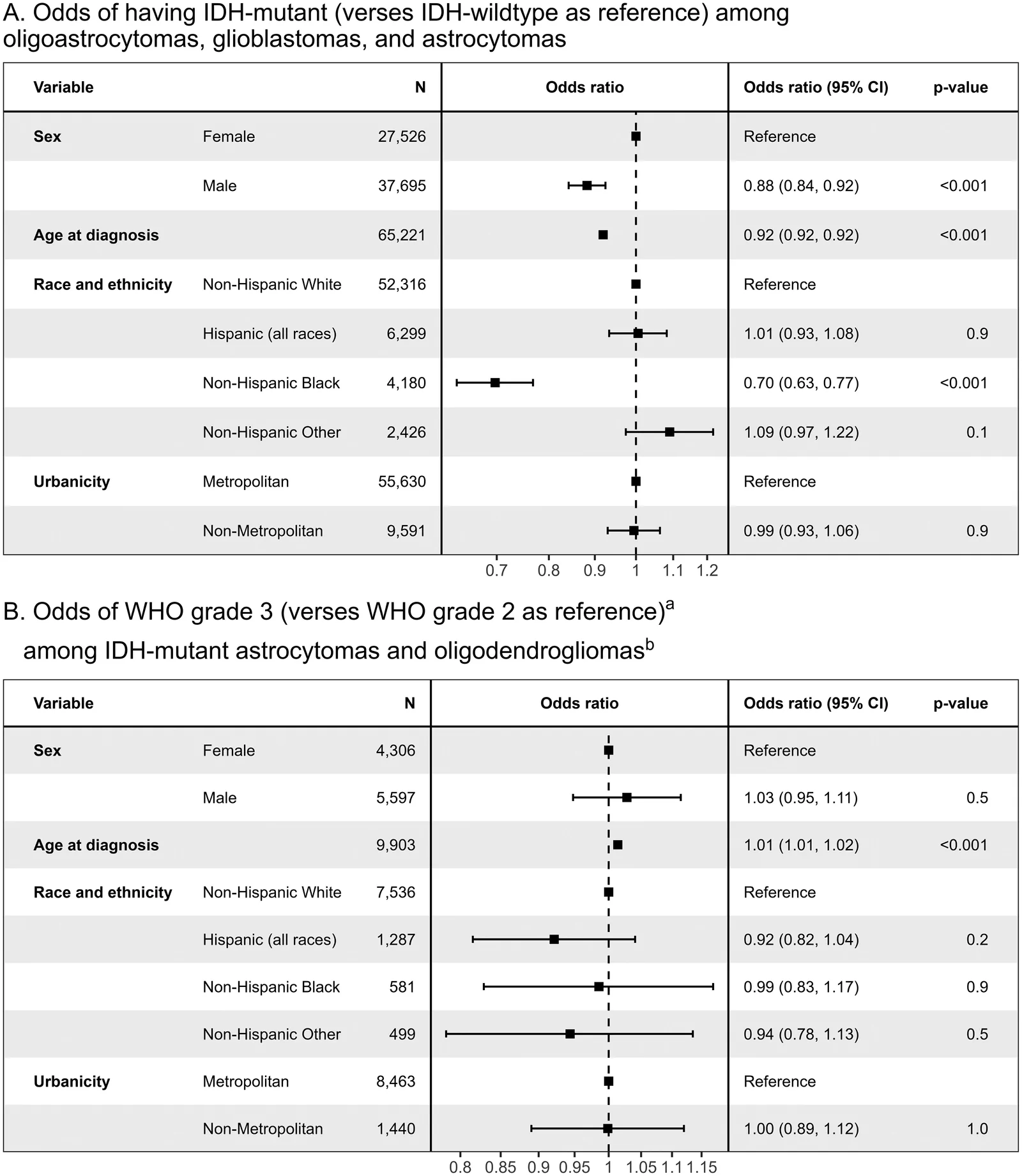
(A) Odds ratios and 95% confidence intervals of multivariable logistic regression to evaluate the impact of selected demographics on the likelihood of IDH-mutant versus IDH-wild-type (reference) among astrocytoma^a^ (including glioblastoma) and (B) odds ratios and 95% CIs of multivariable logistic regression^b^ to evaluate the impact of selected demographics on the likelihood of WHO grade within IDH-mutant astrocytoma and oligodendroglioma. ^a^Grade was recoded using the CBTRUS glioma grade recode (see Price et al.^1^ for additional information). https://doi.org/10.1093/neuonc/noaf194. ^b^Includes brain molecular marker groups Astrocytoma, IDH-mutant (9400/3 and 9401/3) and Oligodendroglioma, IDH-mutant and 1p/19q co-deleted (9450/3 and 94051/3). Significant results with *P*-value <.05. Data Source: Central Brain Tumor Registry of the United States Statistical Report: US Cancer Statistics—CDC’s National Program of Cancer Registries and NCI’s Surveillance, Epidemiology, and End Results Program, 2018-2022.

A separate analysis of IDH-mutant astrocytomas and oligodendrogliomas explored associations between demographic factors and WHO grade at diagnosis (grades 2-3) (Figure 2B). Although higher grades were less frequent for all racial/ethnic groups compared to non-Hispanic White cases, the study did not have sufficient statistical evidence to confidently establish this difference.

### Overall, Median, and Adjusted Survival

In unadjusted survival analysis, IDH-mutant tumors were associated with more favorable 4-year overall and median survival than their IDH-wild-type counterparts, a trend that held across all racial/ethnic groups (Table 2). A prognostic hierarchy was observed among the subtypes, ranging from IDH-wild-type glioblastoma, which had the worst outcomes with median survival times of 12-15 months, to IDH-mutant oligodendroglioma, which had the most favorable prognosis with 4-year overall survival at 88% or higher in all groups. When comparing outcomes within these subtypes, non-Hispanic White cases experienced the lowest 4-year overall survival for IDH-wild-type glioblastoma (14%) and IDH-wild-type astrocytoma (36%). For all other glioma subtypes, non-Hispanic Black cases had the lowest 4-year overall survival rates.

**Table 2.** Three-year overall and median survival for histopathologically confirmed molecularly defined glioma by race and hispanic ethnicity for diagnosis years 2018-2021 with follow-up through 2022

	Astrocytoma, IDH-mutant (9400/3 and 9401/3) (overall median follow-up time 39 months)			Astrocytoma, IDH-wild-type (9400/3 and 9401/3) (overall median follow-up time 39 months)			Oligodendroglioma, IDH-mutant and 1 p/19q co-deleted (9450/3 and 9451/3) (overall median follow-up time 40 months)			Glioblastoma, IDH-wild-type (9440/3) (overall median follow-up time 36 months)			Glioblastoma, IDH-mutant (9445/3) (overall median follow-up time 40 months)		
Characteristic	36 months	Median survival	Overall median follow-up time (months)	36 months	Median survival	Overall median follow-up time (months)	36 months	Median survival	Overall median follow-up time (months)	36 months	Median survival	overall median follow-up time (months)	36 months	Median survival	Overall median follow-up time (months)
Race/ethnicity															
Non-Hispanic White	82% (81%, 84%)	nr (nr, nr)	40	36% (34%, 38%)	21 (20, 22)	40	91% (90%, 92%)	nr (nr, nr)	41	14% (14%, 15%)	12 (12, 12)	37	50% (46%, 54%)	36 (29, 42)	42
Non-Hispanic Black	78% (73%, 84%)	nr (nr, nr)	36	43% (36%, 51%)	27 (20, 37)	39	88% (82%, 94%)	nr (68, nr)	41	18% (16%, 21%)	13 (12, 14)	35	45% (33%, 62%)	23 (18, nr)	40
Non-Hispanic Other	88% (83%, 93%)	nr (nr, nr)	35	58% (50%, 67%)	nr (35, nr)	38	97% (93%, 100%)	nr (66, nr)	38	26% (23%, 29%)	16 (15, 17)	32	58% (43%, 78%)	46 (32, nr)	35
Hispanic (all races)	89% (85%, 92%)	nr (nr, nr)	35	42% (35%, 50%)	27 (21, 35)	37	93% (90%, 96%)	nr (nr ,nr)	38	23% (21%, 25%)	15 (14, 16)	32	55% (42%, 72%)	46 (34, nr)	29

Counts and rates are not presented when fewer than 50 cases were reported for the specific category.

Abbreviation: nr, median survival not reached.

Data Source: National Cancer Database, 2018-2021 with follow-up through December 31, 2022.

To isolate the independent effect of race and ethnicity, multivariable Cox proportional hazards models adjusted for key demographic and clinical factors were used (Figure 3; Table S4). After adjustment, a survival advantage for several racial/ethnic groups compared to non-Hispanic White cases emerged, particularly for glioblastomas. For IDH-wild-type glioblastoma, the risk of death was significantly lower for non-Hispanic Black (HR: 0.86, 95% CI: 0.82-0.91, *P* < .001), Hispanic (HR: 0.75, 95% CI: 0.70-0.79, *P* < .001), and non-Hispanic other cases (HR: 0.67, 95% CI: 0.62-0.72, *P* < .001). A similar advantage was seen for IDH-mutant glioblastoma in Hispanic cases (HR: 0.66, 95% CI: 0.44-0.99, *P* = .046).

**Figure 3.**
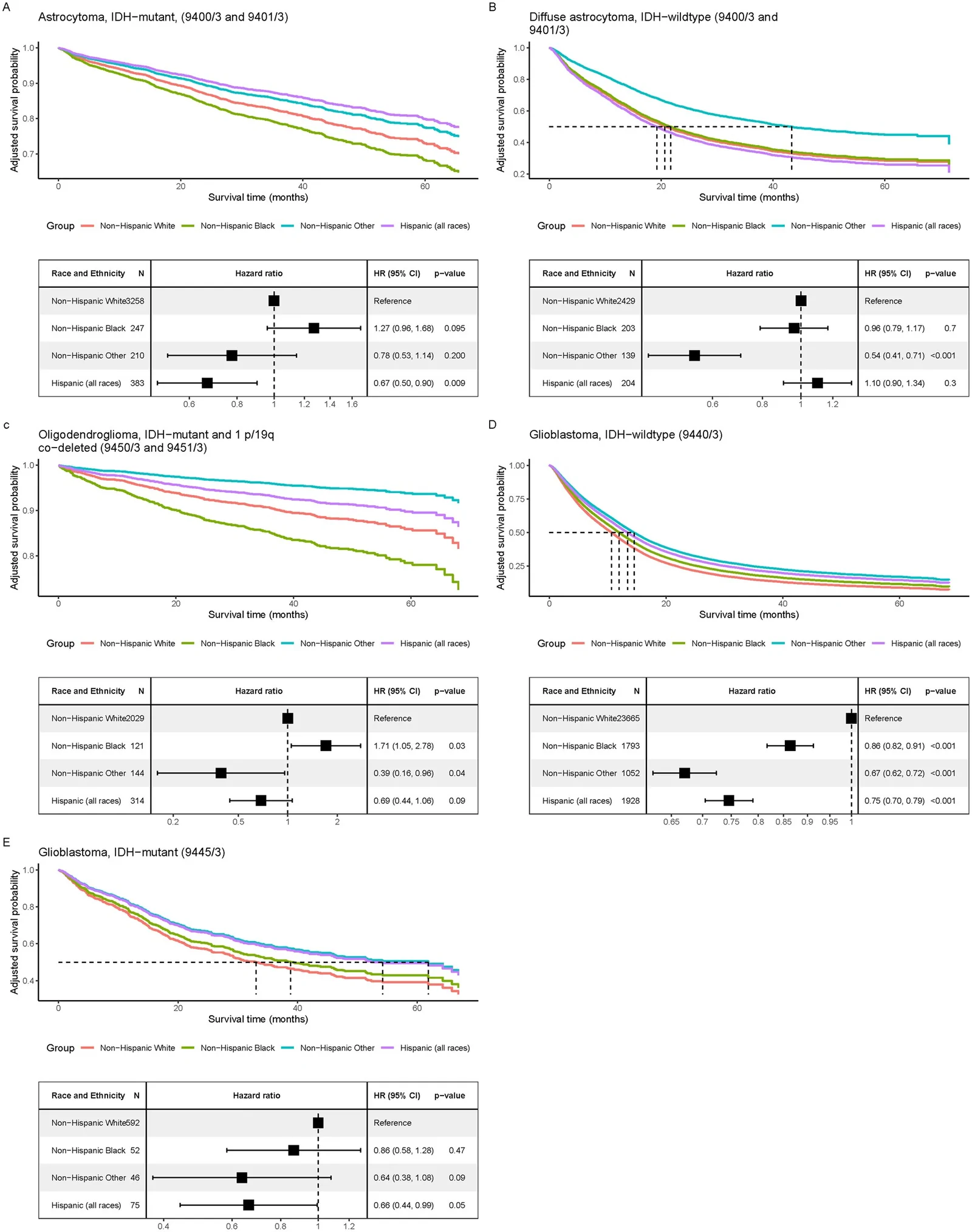
Cox proportional hazards ratios with 95% confidence intervals, adjusted survival curves, and number censored for histopathologically confirmed molecularly defined (A) IDH-wild-type astrocytoma (9400/3 and 9401/3), (B) IDH-mutant astrocytoma (9400/3 and 9401/3), (C) IDH-mutant and 1p/19q co-deleted oligodendroglioma (9450/3 and 9451/3), (D) IDH-wild-type glioblastoma (9440/3), and (E) IDH-mutant glioblastoma (9445/3) tumors for diagnosis years 2018-2021. ^a^Adjusted for age at diagnosis, sex, race/ethnicity, primary payer at diagnosis, WHO grade, extent of surgical resection, and Charlson-Dayo score. ^b^Adjusted for age at diagnosis, race/ethnicity, primary payer at diagnosis, WHO grade, extent of surgical resection, Charlson-Dayo score, and chemotherapy treatment. ^c^Adjusted for age at diagnosis, race/ethnicity, primary payer at diagnosis, WHO grade, extent of surgical resection, Charlson-Dayo score, and urbanicity of residence at diagnosis. ^d^Adjusted for age at diagnosis, sex, race/ethnicity, primary payer at diagnosis, extent of surgical resection, Charlson-Dayo score, radiation treatment, chemotherapy treatment, and urbanicity of residence. ^e^Adjusted for age at diagnosis, race/ethnicity, primary payer at diagnosis, extent of surgical resection, Charlson-Dayo score, radiation treatment, and chemotherapy treatment. Grade was recoded using the CBTRYS glioma grade recode. Database: National Cancer Database, 2018-2021, with follow-up through 2022.

These survival advantages varied among cases with a diagnosis of astrocytoma. A significantly lower risk of death was observed for non-Hispanic other cases with IDH-wild-type astrocytomas (HR: 0.54, 95% CI: 0.41-0.71, *P* < .001) and oligodendrogliomas (HR: 0.39, 95% CI: 0.16-0.96, *P* = .040). However, for cases with oligodendrogliomas, non-Hispanic Black cases alone experienced a significantly higher risk of death (HR: 1.71, 95% CI: 1.05-2.78, *P* = .031). Among IDH-mutant astrocytomas, Hispanic cases had significantly lower risk of death (HR: 0.67, 95% CI: 0.50-0.90, *P* = .009).

Several other clinical and demographic factors were associated with survival across all groups (Table S4). Consistent predictors of increased mortality included older age at diagnosis, higher WHO grade at diagnosis, primary insurance other than commercial, greater extent of surgical resection, and greater comorbidity burden. Among glioblastomas, any chemotherapy or radiation was associated with more favorable survival outcomes (all *P* < .05). Male sex was associated with a higher risk of death for cases with IDH-wild-type glioblastoma (HR: 1.09, 95% CI: 1.06-1.12, *P* < .001) and IDH-mutant astrocytomas (HR: 1.25, 95% CI: 1.08-1.45, *P* = .003). Sex was not significant with all other glioma subtypes in univariable analysis (Table S5) and was thus excluded from multivariable models.

## Discussion

This present work leverages the latest available data from CBTRUS and NCDB to capture nearly all reported molecularly defined adult diffuse-type gliomas in the United States. Consistent with decades of previous research,^24^^,^^25^ we reveal a higher incidence of histopathologically defined glioma among non-Hispanic White individuals with this disparity now shown to persist across distinct molecular entities, including IDH-wild-type and IDH-mutant glioblastoma, astrocytoma, and IDH-mutant and 1p/19q co-deleted oligodendroglioma. Survival following diagnosis of glioma has likewise been shown to be worse among non-Hispanic White cases, even when accounting for treatment type, SES, and access to care. Expanding on previous studies, we reveal higher adjusted mortality for non-Hispanic White cases for both IDH-mutant and -wild-type glioblastoma. Furthermore, we find a lower adjusted risk of death in non-Hispanic other cases with IDH-wild-type astrocytomas, IDH-wild-type glioblastoma, and IDH-mutant and 1p/19q co-deleted oligodendrogliomas, while non-Hispanic Black cases show a uniquely higher risk of death when diagnosed with IDH-mutant and 1p/19q co-deleted oligodendrogliomas. Additional multivariable analysis reveals non-Hispanic Black cases having 23% lower odds of an IDH-wild-type tumor. Although many of the associations between race/ethnicity or BMM subgroup and descriptive variables were statistically significant (*P* < .05), the large sample size allows us to detect very small differences that may not be clinically significant (Cohen’s *w* < 0.1). Overall, the present work validates the longstanding trend of incidence and survival disparities in adult diffuse-type glioma, while revealing distinct associations based on molecular subtype.

While socioeconomic and environmental factors are often invoked in cancer disparities,^13^^,^^26^^,^^27^ the evidence suggests they are insufficient to explain the profound incidence differences in glioma. Non-Hispanic White cases generally have better access to healthcare, which may lead to earlier or more accurate diagnosis of glioma. However, because gliomas are highly symptomatic and rapidly progressive, the likelihood of substantial underdiagnosis in populations with poorer access is considered low.^14^^,^^28-30^ Studies have found that the male-to-female incidence ratio and age at diagnosis are similar across racial/ethnic groups, further suggesting that ascertainment bias is not the primary driver of incidence differences.^14^^,^^31^ We similarly show a male predominance across all molecular subtypes of glioma regardless of race/ethnicity. Multiple large-scale studies report positive associations between higher area-level SES and increased glioma incidence in the United States.^16^^,^^29^^,^^32^^,^^33^ For example, individuals residing in counties in the highest SES quintile have a glioma incidence rate ratio of 1.18 (95% CI: 1.15-1.22) compared to those in the lowest quintile.^16^ This association is most pronounced among non-Hispanic White cases and in urban settings and persists after adjustment for age, sex, and race/ethnicity. Incidence remains high, however, even after adjusting for these factors for non-Hispanic White cases compared to all other groups. We confirm this longstanding observation now among molecularly defined glioma subtypes. Further work is needed to better disentangle these factors.

Environmental exposure to ionizing radiation is the only well-established environmental risk factor for glioma, but such exposures are rare and cannot fully account for population-level differences in incidence.^34-37^ Other proposed environmental risk factors, including occupational exposures, diet, smoking, and alcohol, have not shown consistent associations with gliomagenesis or lifetime risk in large studies.^34-37^ The American Cancer Society notes that exposure to carcinogenic air emissions is up to 50% higher among people experiencing poverty, regardless of race or ethnicity, but for glioma specifically, there is no strong evidence that any known environmental exposures related to SES or neighborhood disadvantage explain the observed differences in glioma incidence.^38^

In the absence of any significant environmental risk factors, the most compelling explanation for these disparities lies in genetic factors. Genome-wide association studies (GWAS) have identified multiple common, low-penetrance single nucleotide polymorphisms that contribute to glioma risk, with the strongest associations observed in populations of European ancestry.^38^^,^^39^ Key risk loci—defined by genome-wide significance (*P* < 5 × 10^−8^) and independent replication—include 5p15.33 telomerase reverse transcriptase (TERT), 8q24.21 CCDC26 Long Non-Coding RNA (CCDC26), 9p21.3 Cyclin-dependent kinase inhibitor 2A/B (CDKN2A/B), 20q13.33 regulator of telomere elongation helicase 1 (RTEL1), and 11q23.3 Pleckstrin Homology Like Domain Family B Member 1 (PHLDB1), among othersothers.^38^ The largest European meta-analysis to date, comprising 12 496 cases and 18 190 controls, identified 13 new risk loci and provided strong evidence that genetic susceptibility to glioblastoma and non-glioblastoma tumors is distinct, reflecting different etiological pathways.^39^ These risk loci display subtype-specific associations when stratified by IDH mutation, TERT promoter status, and 1p/19q codeletion, and GWAS performed within molecularly defined subgroups have identified additional novel susceptibility regions.^38-41^ For example, the TERT locus (rs2736100) is associated with increased risk of glioma, with odds ratios in the range of 1.2-1.3 in European populations.^38-42^ These loci are notable for their involvement in telomere maintenance, cell cycle regulation, and DNA repair. Comparative analyses of European and East Asian GWAS reveal a pattern of partial concordance: *RTEL1* (rs6010620), *TERT* (rs2736100), and *PHLDB1* (rs498872) have been confirmed as glioma risk loci in large Chinese Han GWASs, whereas *CCDC26* (rs4295627) and *CDKN2A/B* (rs4977756) do not demonstrate significant associations in Asian populations, suggesting population-specific genetic architecture.^42^^,^^43^ East Asian GWAS have further identified novel susceptibility loci—including Serine/Threonine Kinase 38 Like (*STK38L,* 12p11.23) and Ras-Related Protein Rab-27A (*RAB27A,* 15q15-21.1)—that have not been detected in European populations, indicating that the full genetic architecture of glioma susceptibility is not entirely captured by European-derived GWAS.^41^^,^^43^ Moreover, admixture mapping studies in African American and Hispanic US populations have provided direct evidence that increased European genetic ancestry is associated with higher glioma risk in multiple glioma subtypes and that specific genomic regions linked to European ancestry confer increased susceptibility.^44-47^ Our data, which confirm the higher incidence in non-Hispanic White cases across all molecular subtypes studied, align squarely with this body of evidence, solidifying the theory that genetic predisposition is a likely primary driver of glioma incidence. Future work is needed to better characterize the varied prevalence of *IDH1/2* mutations and other glioma-specific molecular biomarkers and how they may subsequently influence population-level or regional incidence and survival outcomes within these genetically distinct populations.

### Limitations

There are inherent limitations to the use of cancer registry data. Central cancer registries do not conduct their own central pathology review and only report diagnoses as reported by the diagnosing pathologist at the treating institutions. It has also been shown that survival data are less complete among Hispanic communities due to higher rates of loss to follow-up compared to non-Hispanic communities.^14^^,^^46^ In addition, due to the retrospective nature of this study, analytic choices were not preregistered. The analytic strategy and variable groupings used here reflect prior publications. Furthermore, our study design did not capture certain relevant molecular or clinical details known to impact prognosis including *MGMT* promoter methylation status, *TERT* promoter mutations, or performance status measures that were either not consistently recorded or were not available. Future expansions of the BMM data item, along with longer follow-up windows, are essential to validate our findings and to assess the full effect of molecular stratification on glioma outcomes.

## Conclusions

This study of glioma incidence and survival revealed a higher incidence for non-Hispanic White cases compared to other racial/ethnic groups across all molecular subtypes. Additional differences in survival based on glioma subtype and racial/ethnic background were observed, which underscore the complexity and significance of these molecular classification systems. Future work leveraging new initiatives for the collection of these genetic biomarkers within cancer registry systems is key to elucidating the origins of these disparities and better informing prognosis.

## Supplementary Material

noag157_Supplementary_Data

## Data Availability

Research data are not available for access as it includes sensitive and confidential patient data.
