# Supplementary material for: Differences in primary molecular subtype distribution contribute to racial and ethnic disparities in adult-type diffuse gliomas
Source: Neuro Oncol. 2026 Jul 14;28(11):2728–40. doi: 10.1093/neuonc/noag157 (PMC13731853; doi:10.1093/neuonc/noag157)
Supplement: Supplementary file 1 [file noag157_supplementary_data.zip › Supplementary Tables and Figures_v3.docx]

**Supplementary Methods:**

**Abbreviations:**

95%CI – 95% Confidence Intervals

ANOVA – Analysis of variance

AAAIR – Average age adjusted incidence rates

BMM – Brain Molecular Markers

CBTRUS – Central Brain Tumor Registry of the United States

CCDC26 - CCDC26 Long Non-Coding RNA

CDC – Centers for Disease Control and Prevention

CDKN2A/B - Cyclin-dependent kinase inhibitor 2A/B

GWAS – Genome-wide association studies

HR – Hazard ratios

ICD-O-3 – International Classification of Diseases for Oncology, 3rd edition

*IDH*1/2 – *isocitrate dehydrogenase* 1/2

IRR – Incidence rate ratios

*MGMT* – *O6-methylguanine-DNA methyltransferase*

NAACCR - North American Association of Central Cancer Registries

NCDB – National Cancer Database

NCI – National Cancer Institute

NOS – Not otherwise specified

NPCR - National Program of Cancer Registries

nr – Not reached

OR – Odd ratio

PHLDB1 - Pleckstrin Homology Like Domain Family B Member 1

RAB27A - Ras-Related Protein Rab-27A

RTEL1 - regulator of telomere elongation helicase 1

RUCC – Rural-Urban Continuum Codes

SEER – Surveillance, Epidemiology, and End Results Program

SNPs – Single nucleotide polymorphisms

STK38L - Serine/Threonine Kinase 38 Like

TERT - telomerase reverse transcriptase

U.S. – United States

WHO – World Health Organization

**Supplementary Table 1.** Descriptive table of histopathologically-confirmed, molecularly defined glioma by subtype for diagnosis years 2018-2022

| Characteristic | Astrocytoma, IDH-mutant (9400/3 and 9401/3),  N = 6,095^1^ | Astrocytoma, IDH-wildtype (9400/3 and 9401/3),  N = 4,321^1^ | Glioblastoma, IDH-wildtype (9440/3),  N = 51,548^1^ | Glioblastoma, IDH-mutant (9445/3),  N = 1,460^1^ | Oligodendroglioma, IDH-mutant and 1 p/19q co-deleted (9450/3 and 9451/3), N = 4,478^1^ | Overall,  N = 68,172^1^ | Effect size | p-value^2^ |  |
| --- | --- | --- | --- | --- | --- | --- | --- | --- | --- |
| **Age at Diagnosis^3^** | 37 (29, 49) | 59 (42, 70) | 66 (58, 73) | 48 (35, 63) | 45 (35, 56) | 63 (51, 71) | 0.27 | **<0.001** |  |
| **Age at Diagnosis Group** |  |  |  |  |  |  | 0.53 | **<0.001** |  |
| <50 years | 4,640 (76%) | 1,439 (33%) | 5,552 (11%) | 763 (52%) | 2,886 (61%) | 15,280 (22%) |  |  |  |
| 50+ years | 1,455 (24%) | 2,882 (67%) | 45,996 (89%) | 697 (48%) | 1,862 (39%) | 52,892 (78%) |  |  |  |
| **WHO Grade^4^** |  |  |  |  |  |  | 0.58 | **<0.001** |  |
| Unknown/Other | -- | -- | <16 cases | 54 (3.7%) | -- | 1,026 (1.5%) |  |  |  |
| WHO Grade 2 | 3,345 (55%) | 1,903 (44%) | <16 cases | <16 cases | 2,671 (57%) | 7,919 (12%) |  |  |  |
| WHO Grade 3 | 2,442 (40%) | 1,948 (45%) | <16 cases | <16 cases | 1,883 (40%) | 6,273 (9.2%) |  |  |  |
| WHO Grade 4 | <16 cases | <16 cases | 51,548 (100%) | 1,406 (96%) | <16 cases | 52,954 (78%) |  |  |  |
| **Sex** |  |  |  |  |  |  | 0.02 | **<0.001** |  |
| Female | 2,636 (43%) | 1,955 (45%) | 21,477 (42%) | 600 (41%) | 2,089 (44%) | 28,757 (42%) |  |  |  |
| Male | 3,459 (57%) | 2,366 (55%) | 30,071 (58%) | 860 (59%) | 2,659 (56%) | 39,415 (58%) |  |  |  |
| **Race/Ethnicity** |  |  |  |  |  |  | 0.04 | **<0.001** |  |
| Hispanic (all races) | 766 (13%) | 414 (9.6%) | 4,574 (8.9%) | 169 (12%) | 653 (14%) | 6,576 (9.6%) |  |  |  |
| Non-Hispanic Black | 410 (6.7%) | 304 (7.0%) | 3,205 (6.2%) | 102 (7.0%) | 226 (4.8%) | 4,247 (6.2%) |  |  |  |
| Non-Hispanic Other | 274 (4.5%) | 181 (4.2%) | 1,750 (3.4%) | 69 (4.7%) | 288 (6.1%) | 2,562 (3.8%) |  |  |  |
| Non-Hispanic White | 4,645 (76%) | 3,422 (79%) | 42,019 (82%) | 1,120 (77%) | 3,581 (75%) | 54,787 (80%) |  |  |  |
| **Extent of Surgical Resection** |  |  |  |  |  |  | 0.09 | **<0.001** |  |
| Gross Total Resection | 2,710 (44%) | 1,081 (25%) | 21,386 (41%) | 701 (48%) | 2,355 (50%) | 28,233 (41%) |  |  |  |
| No Surgery or Biopsy Only | 1,410 (23%) | 2,239 (52%) | 14,970 (29%) | -- | -- | 19,776 (29%) |  |  |  |
| Other or Unknown Surgery | 43 (0.7%) | 45 (1.0%) | 368 (0.7%) | <16 cases | -- | 500 (0.7%) |  |  |  |
| Subtotal resection | 1,932 (32%) | 956 (22%) | 14,824 (29%) | 467 (32%) | 1,484 (31%) | 19,663 (29%) |  |  |  |
| **Radiation Treatment** |  |  |  |  |  |  | 0.01 | **0.023** |  |
| No Radiation/Unknown | 5,967 (98%) | 4,198 (97%) | 50,219 (97%) | 1,431 (98%) | 4,646 (98%) | 66,461 (97%) |  |  |  |
| Received any Radiation | 128 (2.1%) | 123 (2.8%) | 1,329 (2.6%) | 29 (2.0%) | 102 (2.1%) | 1,711 (2.5%) |  |  |  |
| **Urbanicity of County of Residence** |  |  |  |  |  |  | 0.01 | 0.2 |  |
| Metropolitan | 4,995 (86%) | 3,515 (85%) | 42,006 (85%) | 1,223 (87%) | 3,891 (86%) | 55,630 (85%) |  |  |  |
| Non-Metropolitan | 847 (14%) | 615 (15%) | 7,299 (15%) | 176 (13%) | 654 (14%) | 9,591 (15%) |  |  |  |
| Unknown | 253 | 191 | 2,243 | 61 | 203 | 2,951 |  |  |  |
| ^1^n (%).  ^2^Cohen’s w effect size and Pearson's Chi-squared test.  ^3^Median (Q1, Q3), partial eta-squared effect size and one-sided ANOVA test.  ^4^ Grade was recoded using the CBTRUS glioma grade recode (see Price, et al 2025 for additional information). <https://doi.org/10.1093/neuonc/noaf194>  --Counts and rates are not presented when fewer than 16 cases were reported for the specific category. The suppressed cases are included in the counts and rates for totals.  **Notes:** Bold denotes statistical significance.  **Data Source:** Central Brain Tumor Registry of the United States Statistical Report: U.S. Cancer Statistics – Centers for Disease Control and Prevention’s National Program of Cancer Registries and National Cancer Institute’s Surveillance, Epidemiology, and End Results Program, 2018-2022.  **Abbreviations:** WHO, World Health Organization | | | | | | | | | |

**Supplementary Table 2.** Five-year total, average annual age-adjusted incidence rates (AAAIR)^1^, and incidence rate ratios (IRR) with 95% confidence intervals (CI) of histopathologically-confirmed, molecularly defined glioma by race and Hispanic ethnicity for diagnosis years 2018-2022

| Brain Molecular Markers | Race/Ethnicity | Count | AAAIR (95% CI) | IRR  (95% CI) | P-Value |  |
| --- | --- | --- | --- | --- | --- | --- |
| Astrocytoma, IDH-mutant (9400/3 and 9401/3) | Non-Hispanic White | 4,645 | 0.49 (0.48,0.51) | Reference |  |  |
|  | Non-Hispanic Black | 410 | 0.19 (0.17,0.21) | 0.39 (0.35,0.43) | **<0.001** |  |
|  | Non-Hispanic Other | 274 | 0.21 (0.19,0.24) | 0.43 (0.38,0.49) | **<0.001** |  |
|  | Hispanic (all races) | 716 | 0.24 (0.22,0.25) | 0.48 (0.44,0.52) | **<0.001** |  |
| Astrocytoma, IDH-wildtype (9400/3 and 9401/3) | Non-Hispanic White | 3,422 | 0.28 (0.27,0.29) | Reference |  |  |
|  | Non-Hispanic Black | 304 | 0.14 (0.12,0.16) | 0.50 (0.44,0.57) | **<0.001** |  |
|  | Non-Hispanic Other | 181 | 0.14 (0.12,0.17) | 0.52 (0.44,0.60) | **<0.001** |  |
|  | Hispanic (all races) | 392 | 0.15 (0.13,0.16) | 0.53 (0.47,0.59) | **<0.001** |  |
| Glioblastoma, IDH-mutant (9445/3) | Non-Hispanic White | 1,120 | 0.10 (0.10,0.11) | Reference |  |  |
|  | Non-Hispanic Black | 102 | 0.05 (0.04,0.06) | 0.47 (0.38,0.58) | **<0.001** |  |
|  | Non-Hispanic Other | 69 | 0.05 (0.04,0.07) | 0.52 (0.40,0.67) | **<0.001** |  |
|  | Hispanic (all races) | 157 | 0.06 (0.05,0.07) | 0.56 (0.47,0.67) | **<0.001** |  |
| Glioblastoma, IDH-wildtype (9440/3) | Non-Hispanic White | 42,019 | 2.87 (2.84,2.90) | Reference |  |  |
|  | Non-Hispanic Black | 3,205 | 1.42 (1.37,1.47) | 0.49 (0.48,0.51) | **<0.001** |  |
|  | Non-Hispanic Other | 1,750 | 1.36 (1.29,1.42) | 0.47 (0.45,0.50) | **<0.001** |  |
|  | Hispanic (all races) | 4,370 | 1.83 (1.78,1.89) | 0.64 (0.62,0.66) | **<0.001** |  |
| Oligodendroglioma, IDH-mutant and 1 p/19q co-deleted (9450/3 and 9451/3) | Non-Hispanic White | 3,581 | 0.36 (0.35,0.37) | Reference |  |  |
|  | Non-Hispanic Black | 226 | 0.11 (0.09,0.12) | 0.30 (0.26,0.35) | **<0.001** |  |
|  | Non-Hispanic Other | 288 | 0.22 (0.20,0.25) | 0.62 (0.55,0.7) | **<0.001** |  |
|  | Hispanic (all races) | 602 | 0.21 (0.19,0.23) | 0.58 (0.53,0.64) | **<0.001** |  |
| ^1^Rates are per 100,000 and are age-adjusted to the 2000 US standard population.  **Notes:** Bold denotes significance. P-values are based on a Wald-type z-test (normal approximation) of the log(IRR) comparing racial and ethnic groups within each tumor type.**Data Source:** Central Brain Tumor Registry of the United States Statistical Report: U.S. Cancer Statistics – Centers for Disease Control and Prevention’s National Program of Cancer Registries and National Cancer Institute’s Surveillance, Epidemiology, and End Results Program, 2018-2022. | | | | | | |

**Supplementary Table 3**. Pairwise comparisons with adjustment for race and ethnicity groups

| **Contrast Group** | **Estimate (95% Cl)** | **P_Value** |
| --- | --- | --- |
| Mutant verses Wildtype^1^ |  |  |
| *(Non-Hispanic White) / Hispanic (all races)* | 0.99 (0.90-1.10) | 0.999 |
| *(Non-Hispanic White) / (Non-Hispanic Black)* | 1.43 (1.26-1.63) | **<0.001** |
| *(Non-Hispanic White) / (Non-Hispanic Other)* | 0.92 (0.79-1.06) | 0.429 |
| *Hispanic (all races) / (Non-Hispanic Black)* | 1.44 (1.24-1.68) | **<0.001** |
| *Hispanic (all races) / (Non-Hispanic Other)* | 0.92 (0.78-1.09) | 0.6 |
| *(Non-Hispanic Black) / (Non-Hispanic Other)* | 0.64 (0.53-0.77) | **<0.001** |
| Grade 1 verses 3^1^ |  |  |
| *(Non-Hispanic White) / Hispanic (all races)* | 1.09 (0.92-1.27) | 0.552 |
| *(Non-Hispanic White) / (Non-Hispanic Black)* | 1.01 (0.81-1.27) | 0.998 |
| *(Non-Hispanic White) / (Non-Hispanic Other)* | 1.06 (0.83-1.35) | 0.928 |
| *Hispanic (all races) / (Non-Hispanic Black)* | 0.93 (0.72-1.22) | 0.913 |
| *Hispanic (all races) / (Non-Hispanic Other)* | 0.98 (0.74-1.29) | 0.996 |
| *(Non-Hispanic Black) / (Non-Hispanic Other)* | 1.04 (0.76-1.44) | 0.986 |
| Astrocytoma, IDH-mutant, (9400/3 and 9401/3) ^2^ |  |  |
| *(Non-Hispanic White) / Hispanic (all races)* | 0.79 (0.54-1.15) | 0.284 |
| *(Non-Hispanic White) / (Non-Hispanic Black)* | 1.29 (0.76-2.17) | 0.401 |
| *(Non-Hispanic White) / (Non-Hispanic Other)* | 1.49 (1.00-2.24) | **0.044** |
| *Hispanic (all races) / (Non-Hispanic Black)* | 1.64 (0.87-3.07) | 0.152 |
| *Hispanic (all races) / (Non-Hispanic Other)* | 1.90 (1.12-3.22) | **0.008** |
| *(Non-Hispanic Black) / (Non-Hispanic Other)* | 1.16 (0.61-2.20) | 0.542 |
| Diffuse astrocytoma, IDH-wildtype (9400/3 and 9401/3)^2^ |  |  |
| *(Non-Hispanic White) / Hispanic (all races)* | 1.04 (0.80-1.35) | 0.941 |
| *(Non-Hispanic White) / (Non-Hispanic Black)* | 1.85 (1.29-2.65) | **<0.001** |
| *(Non-Hispanic White) / (Non-Hispanic Other)* | 0.91 (0.70-1.18) | 0.941 |
| *Hispanic (all races) / (Non-Hispanic Black)* | 1.78 (1.15-2.74) | **0.002** |
| *Hispanic (all races) / (Non-Hispanic Other)* | 0.87 (0.61-1.24) | 0.941 |
| *(Non-Hispanic Black) / (Non-Hispanic Other)* | 0.49 (0.32-0.76) | **<0.001** |
| Oligodendroglioma, IDH-mutant and 1 p/19q co-deleted (9450/3 and 9451/3) ^2^ |  |  |
| *(Non-Hispanic White) / Hispanic (all races)* | 0.59 (0.30-1.13) | 0.125 |
| *(Non-Hispanic White) / (Non-Hispanic Black)* | 2.55 (0.77-8.48) | 0.125 |
| *(Non-Hispanic White) / (Non-Hispanic Other)* | 1.46 (0.81-2.63) | 0.186 |
| *Hispanic (all races) / (Non-Hispanic Black)* | 4.35 (1.14-16.66) | **0.023** |
| *Hispanic (all races) / (Non-Hispanic Other)* | 2.49 (1.07-5.75) | **0.023** |
| *(Non-Hispanic Black) / (Non-Hispanic Other)* | 0.57 (0.16-2.09) | 0.255 |
| Glioblastoma, IDH-mutant (9445/3) ^2^ |  |  |
| *(Non-Hispanic White) / Hispanic (all races)* | 1.16 (0.68-1.97) | 1.00 |
| *(Non-Hispanic White) / (Non-Hispanic Black)* | 1.57 (0.77-3.20) | 0.467 |
| *(Non-Hispanic White) / (Non-Hispanic Other)* | 1.51 (0.88-2.61) | 0.277 |
| *Hispanic (all races) / (Non-Hispanic Black)* | 1.36 (0.58-3.19) | 1.00 |
| *Hispanic (all races) / (Non-Hispanic Other)* | 1.31 (0.63-2.70) | 1.00 |
| *(Non-Hispanic Black) / (Non-Hispanic Other)* | 0.96 (0.40-2.28) | 1.00 |
| Glioblastoma, IDH-wildtype (9440/3) ^2^ |  |  |
| *(Non-Hispanic White) / Hispanic (all races)* | 1.16 (1.07-1.25) | **<0.001** |
| *(Non-Hispanic White) / (Non-Hispanic Black)* | 1.49 (1.35-1.65) | **<0.001** |
| *(Non-Hispanic White) / (Non-Hispanic Other)* | 1.34 (1.24-1.45) | **<0.001** |
| *Hispanic (all races) / (Non-Hispanic Black)* | 1.29 (1.14-1.46) | **<0.001** |
| *Hispanic (all races) / (Non-Hispanic Other)* | 1.16 (1.05-1.28) | **<0.001** |
| *(Non-Hispanic Black) / (Non-Hispanic Other)* | 0.90 (0.79-1.02) | **0.024** |
| Logistic regression with tukey adjustment. Estimate is Odds Ratio with 95% Confidence Interval. Data from the Central Brain Tumor Registry of the United States Statistical Report: U.S. Cancer Statistics – Centers for Disease Control and Prevention’s National Program of Cancer Registries and National Cancer Institute’s Surveillance, Epidemiology, and End Results Program, 2018-2022.  ^2^ Cox proportional hazards ratios with holm adjustment. Estimate is Hazards Ratio with 95% Confidence Interval. Data from the National Cancer Database, 2018-2022.  **Notes:** Bold denotes significance assessed at p-value<0.05. | | |

**Supplementary Table 4**. Multivariable^1^ Cox proportionalhazards ratios (HR) and 95% confidence intervals **(95%CI)** for histopathologically-confirmed molecularly defined glioma for diagnosis years 2018-2022

|  | Astrocytoma, IDH-mutant (9400/3 and 9401/3) | | | Astrocytoma, IDH-wildtype (9400/3 and 9401/3) | | | Oligodendroglioma, IDH-mutant and 1 p/19q co-deleted (9450/3 and 9451/3)^2^ | | | Glioblastoma, IDH-wildtype (9440/3) | | | Glioblastoma, IDH-mutant (9445/3) | | |
| --- | --- | --- | --- | --- | --- | --- | --- | --- | --- | --- | --- | --- | --- | --- | --- |
| **Characteristic** | **N** | **HR**^1^ **(95%CI)** | **p-value** | **N** | **HR**^1^ **(95%CI)** | **p-value** | **N** | **HR**^1^ **(95%CI)** | **p-value** | **N** | **HR**^1^ **(95%CI)** | **p-value** | **N** | **HR**^1^ **(95%CI)** | **p-value** |
| **Age at diagnosis** | 4,098 | 1.03 (1.03, 1.04) | **<0.001** | 2,975 | 1.03 (1.02, 1.03) | **<0.001** | 2,608 | 1.05 (1.04, 1.07) | **<0.001** | 28,438 | 1.02 (1.02, 1.02) | **<0.001** | 765 | 1.04 (1.03, 1.05) | **<0.001** |
| **Race/Ethnicity** |  |  |  |  |  |  |  |  |  |  |  |  |  |  |  |
| Non-Hispanic White | 3,258 | Reference |  | 2,429 | Reference |  | 2,029 | Reference |  | 23,665 | Reference |  | 592 | Reference |  |
| Non-Hispanic Black | 247 | 1.27 (0.96, 1.68) | 0.095 | 203 | 0.96 (0.79, 1.17) | 0.689 | 121 | 1.71 (1.05, 2.78) | **0.031** | 1,793 | 0.86 (0.82, 0.91) | **<0.001** | 52 | 0.86 (0.58, 1.28) | 0.471 |
| Non-Hispanic Other | 210 | 0.78 (0.53, 1.14) | 0.200 | 139 | 0.54 (0.41, 0.71) | **<0.001** | 144 | 0.39 (0.16, 0.96) | **0.040** | 1,052 | 0.67 (0.62, 0.72) | **<0.001** | 46 | 0.64 (0.38, 1.08) | 0.093 |
| Hispanic (all races) | 383 | 0.67 (0.50, 0.90) | **0.009** | 204 | 1.10 (0.90, 1.34) | 0.343 | 314 | 0.69 (0.44, 1.06) | 0.093 | 1,928 | 0.75 (0.70, 0.79) | **<0.001** | 75 | 0.66 (0.44, 0.99) | **0.046** |
| **WHO grade^3^** |  |  |  |  |  |  |  |  |  |  |  |  |  |  |  |
| WHO grade 2 | 2,180 | Reference |  | 1,453 | Reference |  | 1,479 | Reference |  |  | Not included |  |  | Not included |  |
| WHO grade 3 | 1,918 | 1.92 (1.65, 2.22) | **<0.001** | 1,522 | 1.58 (1.44, 1.74) | **<0.001** | 1,129 | 2.16 (1.66, 2.82) | **<0.001** |  |  |  |  |  |  |
| **Primary Payer at Diagnosis** |  |  |  |  |  |  |  |  |  |  |  |  |  |  |  |
| Commercial | 2,862 | Reference |  | 1,550 | Reference |  | 1,768 | Reference |  | 12,244 | Reference |  | 471 | Reference |  |
| Medicare | 323 | 2.01 (1.61, 2.51) | **<0.001** | 973 | 1.03 (0.91, 1.17) | 0.654 | 251 | 1.18 (0.81, 1.74) | 0.392 | 12,868 | 1.15 (1.11, 1.19) | **<0.001** | 149 | 1.25 (0.92, 1.71) | 0.148 |
| Medicaid | 694 | 1.10 (0.88, 1.37) | 0.404 | 334 | 1.24 (1.04, 1.47) | **0.016** | 446 | 1.73 (1.22, 2.45) | **0.002** | 2,257 | 1.08 (1.02, 1.14) | **0.005** | 110 | 1.00 (0.72, 1.39) | 0.987 |
| Not Insured | 167 | 1.93 (1.36, 2.73) | **<0.001** | 87 | 1.40 (1.06, 1.84) | **0.018** | 123 | 3.61 (2.30, 5.67) | **<0.001** | 746 | 1.16 (1.07, 1.27) | **<0.001** | -- | -- | -- |
| Insurance Status Unknown | 52 | 1.11 (0.55, 2.23) | 0.780 | 31 | 1.28 (0.78, 2.10) | 0.337 | 20 | 0.00 (0.00, Inf) | 0.991 | 323 | 1.11 (0.98, 1.25) | 0.105 | <10 cases | -- | -- |
| **Extent of resection** |  |  |  |  |  |  |  |  |  |  |  |  |  |  |  |
| Gross-total | 1,810 | Reference |  | 721 | Reference |  | 1,347 | Reference |  | 11,734 | Reference |  | 376 | Reference |  |
| Subtotal | 1,339 | 1.48 (1.23, 1.79) | **<0.001** | 673 | 1.37 (1.17, 1.61) | **<0.001** | 950 | 1.43 (1.09, 1.87) | **0.009** | 8,528 | 1.26 (1.22, 1.30) | **<0.001** | 259 | 1.16 (0.92, 1.46) | 0.219 |
| Other or excisional biopsy only | 398 | 2.08 (1.63, 2.65) | **<0.001** | 402 | 1.99 (1.68, 2.36) | **<0.001** | 209 | 1.26 (0.79, 2.02) | 0.336 | 3,120 | 1.32 (1.26, 1.37) | **<0.001** | 60 | 1.28 (0.87, 1.88) | 0.218 |
| None | 551 | 3.69 (3.03, 4.50) | **<0.001** | 1,179 | 2.82 (2.46, 3.23) | **<0.001** | 102 | 1.61 (0.96, 2.67) | 0.069 | 5,056 | 2.04 (1.97, 2.12) | **<0.001** | 70 | 2.07 (1.49, 2.88) | **<0.001** |
| **Charlson-Dayo score** |  |  |  |  |  |  |  |  |  |  |  |  |  |  |  |
| 0 | 3,521 | Reference |  | 2,304 | Reference |  | 2,186 | Reference |  | 20,260 | Reference |  | 625 | Reference |  |
| 1 | 381 | 1.12 (0.90, 1.41) | 0.309 | 391 | 1.13 (0.99, 1.29) | 0.074 | 277 | 1.25 (0.88, 1.78) | 0.206 | 4,353 | 1.16 (1.12, 1.21) | **<0.001** | 77 | 1.09 (0.79, 1.52) | 0.589 |
| 2 | 137 | 1.25 (0.90, 1.73) | 0.184 | 162 | 1.44 (1.20, 1.73) | **<0.001** | 93 | 1.35 (0.73, 2.49) | 0.340 | 2,176 | 1.26 (1.20, 1.33) | **<0.001** | 39 | 1.74 (1.18, 2.57) | **0.005** |
| 3+ | 59 | 1.54 (1.02, 2.32) | **0.038** | 118 | 1.90 (1.53, 2.35) | **<0.001** | 52 | 2.98 (1.83, 4.87) | **<0.001** | 1,649 | 1.32 (1.25, 1.40) | **<0.001** | 24 | 1.33 (0.77, 2.30) | 0.302 |
| **Radiation received, any** |  | Not included |  |  | Not included |  |  | Not included |  |  |  |  |  |  |  |
| No |  |  |  |  |  |  |  |  |  | 6,340 | Reference |  | 115 | Reference |  |
| Yes |  |  |  |  |  |  |  |  |  | 22,098 | 0.56 (0.53, 0.58) | **<0.001** | 650 | 0.56 (0.37, 0.84) | **0.006** |
| **Chemotherapy received, any** |  | Not included |  |  |  |  |  | Not included |  |  |  |  |  |  |  |
| No |  |  |  | 839 | Reference |  |  |  |  | 7,443 | Reference |  | 120 | Reference |  |
| Yes |  |  |  | 2,136 | 0.63 (0.56, 0.70) | **<0.001** |  |  |  | 20,995 | 0.57 (0.54, 0.59) | **<0.001** | 645 | 0.47 (0.31, 0.70) | **<0.001** |
| **Urbanicity** |  | Not included |  |  | Not included |  |  |  |  |  |  |  |  | Not included |  |
| Non-Metropolitan |  |  |  |  |  |  | 447 | Reference |  | 4,685 | Reference |  |  |  |  |
| Metropolitan |  |  |  |  |  |  | 2,161 | 0.87 (0.65, 1.18) | 0.374 | 23,753 | 0.93 (0.90, 0.96) | **<0.001** |  |  |  |
| **Sex** |  |  |  |  | Not included |  |  | Not included |  |  |  |  |  | Not included |  |
| Female | 1,718 | Reference |  |  |  |  |  |  |  | 11,769 | Reference |  |  |  |  |
| Male | 2,380 | 1.25 (1.08, 1.45) | **0.003** |  |  |  |  |  |  | 16,669 | 1.09 (1.06, 1.12) | **<0.001** |  |  |  |
| ^1^Only variables that were significant in univariable analyses were included in multivariable models.  ^2^Oligodendroglioma, NOS histopathology code 9450/3 that were grade 1 or grade 4 were excluded from analysis.  ^3^Grade was recoded using the CBTRUS glioma grade recode (see Price, et al 2025 for additional information). <https://doi.org/10.1093/neuonc/noaf194>  --Counts and rates are not presented when fewer than 10 cases were reported for the specific category. The suppressed cases are included in the counts and rates for totals.  **Notes:** Bold denotes significance.  **Data Source:** National Cancer Database, 2018-2022.  **Abbreviations:** WHO, World Health Organization. | | | | | | | | | | | | | | | |

**Supplementary Table 5**. Univariable Cox proportional hazards ratios (HR) and 95% confidence intervals (95% CI) for histopathologically-confirmed molecularly defined glioma for diagnosis years 2018-2022

|  | Glioblastoma, IDH-wildtype (9440/3) | | | Oligodendroglioma, IDH-mutant and 1 p/19q co-deleted (9450/3 and 9451/3)^1^ | | | Astrocytoma, IDH-mutant, (9400/3 and 9401/3) | | | Diffuse astrocytoma, IDH-wildtype (9400/3 and 9401/3) | | | Glioblastoma, IDH-mutant (9445/3) | | |
| --- | --- | --- | --- | --- | --- | --- | --- | --- | --- | --- | --- | --- | --- | --- | --- |
| **Characteristic** | **N** | **HR (95%CI)** | **p-value** | **N** | **HR (95%CI)** | **p-value** | **N** | **HR (95%CI)** | **p-value** | **N** | **HR (95%CI)** | **p-value** | **N** | **HR (95%CI)** | **p-value** |
| **Age at diagnosis** | 30,504 | 1.03 (1.03, 1.03) | **<0.001** | 2,778 | 1.06 (1.05, 1.07) | **<0.001** | 4,103 | 1.05 (1.05, 1.06) | **<0.001** | 3,046 | 1.04 (1.03, 1.04) | **<0.001** | 791 | 1.05 (1.05, 1.06) | **<0.001** |
| **Primary insurance payer** |  |  |  |  |  |  |  |  |  |  |  |  |  |  |  |
| Commercial | 13,185 | Reference |  | 1,907 | Reference |  | 2,864 | Reference |  | 1,584 | Reference |  | 483 | Reference |  |
| Medicare | 13,754 | 1.77 (1.72, 1.82) | **<0.001** | 264 | 4.04 (3.00, 5.45) | **<0.001** | 325 | 5.73 (4.80, 6.82) | **<0.001** | 998 | 2.29 (2.07, 2.53) | **<0.001** | 159 | 3.95 (3.15, 4.95) | **<0.001** |
| Medicaid | 2,400 | 1.08 (1.03, 1.14) | **0.003** | 456 | 1.46 (1.04, 2.05) | **0.028** | 694 | 0.99 (0.80, 1.23) | 0.923 | 342 | 1.00 (0.84, 1.17) | 0.954 | 112 | 1.12 (0.82, 1.55) | 0.473 |
| Not Insured | 808 | 1.21 (1.11, 1.32) | **<0.001** | 129 | 3.06 (1.97, 4.76) | **<0.001** | 168 | 1.73 (1.23, 2.44) | **0.002** | 89 | 1.58 (1.21, 2.07) | **<0.001** | -- | -- | -- |
| Insurance Status Unknown | 357 | 1.39 (1.23, 1.56) | **<0.001** | 22 | 0.00 (0.00, Inf) | 0.988 | 52 | 1.00 (0.50, 2.02) | 0.993 | 33 | 1.25 (0.77, 2.02) | 0.369 | <10 cases | -- | -- |
| **Sex** |  |  |  |  |  |  |  |  |  |  |  |  |  |  |  |
| Female | 12,605 | Reference |  | 1,195 | Reference |  | 1,722 | Reference |  | 1,360 | Reference |  | 320 | Reference |  |
| Male | 17,899 | 1.05 (1.02, 1.08) | **<0.001** | 1,583 | 1.16 (0.90, 1.48) | 0.246 | 2,381 | 1.19 (1.03, 1.38) | **0.020** | 1,686 | 1.04 (0.95, 1.15) | 0.359 | 471 | 1.10 (0.90, 1.35) | 0.343 |
| **Race-ethnicity** |  |  |  |  |  |  |  |  |  |  |  |  |  |  |  |
| Non-Hispanic White | 25,416 | Reference |  | 2,179 | Reference |  | 3,261 | Reference |  | 2,475 | Reference |  | 611 | Reference |  |
| Non-Hispanic Black | 1,890 | 0.90 (0.85, 0.95) | **<0.001** | 126 | 1.48 (0.91, 2.39) | 0.111 | 247 | 1.26 (0.96, 1.66) | 0.098 | 211 | 0.87 (0.72, 1.05) | 0.151 | 53 | 1.11 (0.75, 1.63) | 0.608 |
| Non-Hispanic Other | 1,129 | 0.69 (0.64, 0.74) | **<0.001** | 156 | 0.32 (0.13, 0.79) | **0.013** | 210 | 0.70 (0.48,1.04) | 0.075 | 147 | 0.51 (0.39, 0.66) | **<0.001** | 47 | 0.63 (0.38, 1.04) | 0.069 |
| Hispanic (all races) | 2,069 | 0.76 (0.72, 0.80) | **<0.001** | 317 | 0.79 (0.52, 1.20) | 0.270 | 385 | 0.67 (0.50, 0.91) | **0.009** | 213 | 0.85 (0.71, 1.03) | 0.103 | 80 | 0.71 (0.48, 1.05) | 0.083 |
| **Tumor size** |  |  |  |  |  |  |  |  |  |  |  |  |  |  |  |
| <50 mm | 15,454 | Reference |  | 1,041 | Reference |  | 1,678 | Reference |  | 1,386 | Reference |  | 294 | Reference |  |
| >200 mm | -- | -- | -- | <10 cases | -- | -- | <10 cases | -- | -- | <10 cases | -- | -- | <10 cases | -- | -- |
| 50-200 mm | 9,693 | 1.09 (1.06, 1.12) | **<0.001** | 1,044 | 1.46 (1.09, 1.94) | **0.010** | 1,397 | 0.97 (0.81, 1.16) | 0.705 | 498 | 0.74 (0.64, 0.86) | **<0.001** | 363 | 0.79 (0.63, 0.98) | **0.030** |
| No tumor | <10 cases | -- | -- | <10 cases | -- | -- | <10 cases | -- | -- | <10 cases | -- | -- | <10 cases | -- | -- |
| Unknown/Missing | 5,300 | 1.07 (1.03, 1.11) | **<0.001** | 691 | 1.40 (1.02, 1.93) | **0.039** | 1,024 | 1.82 (1.54, 2.16) | **<0.001** | 1,162 | 1.19 (1.08, 1.32) | **<0.001** | 133 | 0.86 (0.65, 1.16) | 0.326 |
| **Charlson-Dayo score** |  |  |  |  |  |  |  |  |  |  |  |  |  |  |  |
| 0 | 21,738 | Reference |  | 2,335 | Reference |  | 3,522 | Reference |  | 2,348 | Reference |  | 639 | Reference |  |
| 1 | 4,661 | 1.29 (1.24, 1.33) | **<0.001** | 292 | 1.71 (1.22, 2.39) | **0.002** | 384 | 1.51 (1.21, 1.89) | **<0.001** | 410 | 1.44 (1.27, 1.64) | **<0.001** | 84 | 1.37 (1.00, 1.86) | **0.050** |
| 2 | 2,329 | 1.45 (1.38, 1.52) | **<0.001** | 97 | 1.65 (0.92, 2.95) | 0.093 | 138 | 1.72 (1.24, 2.37) | **0.001** | 166 | 1.96 (1.63, 2.34) | **<0.001** | 42 | 2.08 (1.43, 3.02) | **<0.001** |
| 3+ | 1,776 | 1.70 (1.61, 1.79) | **<0.001** | 54 | 6.18 (3.94, 9.71) | **<0.001** | 59 | 3.29 (2.20, 4.91) | **<0.001** | 122 | 3.19 (2.60, 3.91) | **<0.001** | 26 | 1.91 (1.15, 3.16) | **0.012** |
| **Extent of resection** |  |  |  |  |  |  |  |  |  |  |  |  |  |  |  |
| Gross-total | 12,565 | Reference |  | 1,425 | Reference |  | 1,810 | Reference |  | 736 | Reference |  | 389 | Reference |  |
| Subtotal | 9,195 | 1.28 (1.24, 1.32) | **<0.001** | 1,013 | 1.31 (1.01, 1.70) | **0.045** | 1,339 | 1.50 (1.24, 1.82) | **<0.001** | 691 | 1.41 (1.21, 1.65) | **<0.001** | 264 | 1.31 (1.04, 1.65) | **0.020** |
| Other or excisional biopsy only | 3,341 | 1.44 (1.38, 1.50) | **<0.001** | 226 | 1.36 (0.87, 2.11) | 0.174 | 398 | 2.44 (1.92, 3.11) | **<0.001** | 410 | 2.31 (1.95, 2.72) | **<0.001** | 66 | 1.38 (0.96, 1.99) | 0.082 |
| None | 5,384 | 2.34 (2.26, 2.43) | **<0.001** | 112 | 2.27 (1.39, 3.68) | **<0.001** | 551 | 4.91 (4.06, 5.95) | **<0.001** | 1,201 | 3.39 (2.97, 3.87) | **<0.001** | 72 | 4.14 (3.04, 5.63) | **<0.001** |
| **Radiation received, any** |  |  |  |  |  |  |  |  |  |  |  |  |  |  |  |
| No | 6,726 | Reference |  | 1,002 | Reference |  | 1,126 | Reference |  | 749 | Reference |  | 119 | Reference |  |
| Yes | 23,177 | 0.33 (0.32, 0.34) | **<0.001** | 1,723 | 1.10 (0.85, 1.42) | 0.478 | 2,860 | 1.02 (0.87, 1.20) | 0.803 | 2,226 | 0.92 (0.83, 1.03) | 0.167 | 652 | 0.27 (0.21, 0.34) | **<0.001** |
| **Chemotherapy received, any** |  |  |  |  |  |  |  |  |  |  |  |  |  |  |  |
| No | 7,889 | Reference |  | 979 | Reference |  | 1,223 | Reference |  | 842 | Reference |  | 123 | Reference |  |
| Yes | 22,078 | 0.35 (0.34, 0.36) | **<0.001** | 1,729 | 0.83 (0.65, 1.07) | 0.149 | 2,781 | 0.88 (0.76, 1.03) | 0.119 | 2,137 | 0.79 (0.71, 0.87) | **<0.001** | 647 | 0.26 (0.20, 0.33) | **<0.001** |
| **Urbanicity** |  |  |  |  |  |  |  |  |  |  |  |  |  |  |  |
| Non-Metropolitan | 4,824 | Reference |  | 447 | Reference |  | 651 | Reference |  | 503 | Reference |  | 100 | Reference |  |
| Metropolitan | 24,340 | 0.89 (0.86, 0.92) | **<0.001** | 2,163 | 0.73 (0.54, 0.98) | **0.034** | 3,200 | 0.93 (0.76, 1.12) | 0.427 | 2,403 | 0.94 (0.83, 1.07) | 0.343 | 642 | 1.07 (0.79, 1.46) | 0.648 |
| **WHO grade^2^** |  | Not applicable |  |  |  |  |  |  |  |  |  |  |  | Not applicable |  |
| Grade 2 |  |  |  | 1,588 | Reference |  | 2,183 | Reference |  | 1,486 | Reference |  |  |  |  |
| Grade 3 |  |  |  | 1,190 | 2.53 (1.97, 3.25) | **<0.001** | 1,920 | 1.85 (1.59, 2.14) | **<0.001** | 1,560 | 1.54 (1.40, 1.69) | **<0.001** |  |  |  |
| ^1^Oligodendroglioma, NOS histopathology code 9450/3 that were grade 1 or grade 4 were excluded from analysis.  ^2^Grade was recoded using the CBTRUS glioma grade recode (see Price, et al 2025 for additional information). <https://doi.org/10.1093/neuonc/noaf194>  --Counts and rates are not presented when fewer than 10 cases were reported for the specific category. The suppressed cases are included in the counts and rates for totals.  **Notes:** Bold denotes significance.  **Data Source:** National Cancer Database, 2018-2022.  **Abbreviations:** WHO, World Health Organization. | | | | | | | | | | | | | | | |

Supplementary Figure 1. Proportion of IDH-wildtype and -mutant histopathologically-confirmed molecularly defined astrocytoma and glioblastoma for diagnosis years 2018-2022.

Supplementary Figure 2. Kaplan-Meier curves by brain molecular marker subtype stratified by race/ethnicity (data from the National Cancer Database)

P-values represent log-rank tests comparing survival distributions across racial and ethnic groups.

Data source: National Cancer Database, 2018–2021, with follow-up through 2022.
